# A CRISPR/Cas9-riboswitch-Based Method for Downregulation of Gene Expression in *Trypanosoma cruzi*

**DOI:** 10.3389/fcimb.2020.00068

**Published:** 2020-02-27

**Authors:** Noelia Lander, Teresa Cruz-Bustos, Roberto Docampo

**Affiliations:** ^1^Center for Tropical and Emerging Global Diseases, University of Georgia, Athens, GA, United States; ^2^Department of Cellular Biology, University of Georgia, Athens, GA, United States

**Keywords:** acidocalcisome, CRISPR/Cas9, *glmS*, GP72, riboswitch, *Trypanosoma cruzi*, vacuolar proton pyrophosphatase

## Abstract

Few genetic tools were available to work with *Trypanosoma cruzi* until the recent introduction of the CRISPR/Cas9 technique for gene knockout, gene knock-in, gene complementation, and endogenous gene tagging. Riboswitches are naturally occurring self-cleaving RNAs (ribozymes) that can be ligand-activated. Results from our laboratory recently demonstrated the usefulness of the *glmS* ribozyme from *Bacillus subtilis*, which has been shown to control reporter gene expression in response to exogenous glucosamine, for gene silencing in *Trypanosoma brucei*. In this work we used the CRISPR/Cas9 system for endogenously tagging *T. cruzi* glycoprotein 72 (*TcGP72*) and vacuolar proton pyrophosphatase (*TcVP1*) with the active (*glmS*) or inactive (*M9*) ribozyme. Gene tagging was confirmed by PCR and protein downregulation was verified by western blot analyses. Further phenotypic characterization was performed by immunofluorescence analysis and quantification of growth *in vitro*. Our results indicate that the method was successful in silencing the expression of both genes without the need of glucosamine in the medium, suggesting that *T. cruzi* produces enough levels of endogenous glucosamine 6-phosphate to stimulate the *glmS* ribozyme activity under normal growth conditions. This method could be useful to obtain knockdowns of essential genes in *T. cruzi* and to validate potential drug targets in this parasite.

## Introduction

Infection by *Trypanosoma cruzi* is the main cause of congestive heart failure in Latin America (Rassi et al., 2000). The disease affects 8–10 million people in the Americas. The FDA approval of a test for these parasites in donated blood (Kessler et al., 2013) emphasizes the relevance of this parasite to human health in the United States, where physicians are largely unaware of the cardiac symptoms of chronic *T. cruzi* infection. Treatment of Chagas disease is limited to drugs with relatively high toxicity and partial efficacy (Urbina and Docampo, 2003). The study of metabolic pathways in these parasites that could be important for their viability but that do not affect their host could result in the finding of specific inhibitors to control the parasites without altering the hosts. A drawback for these studies in *T. cruzi* has been the lack of genetic tools, such as inducible downregulation, which are essential for the demonstration of the essentiality of these metabolic pathways and for the validation of new drug targets.

Few genetic tools were available to work with *T. cruzi* (Docampo, 2011; Burle-Caldas Gde et al., 2015) until the recent introduction of the CRISPR/Cas9 technique for gene knockout (Peng et al., 2014; Lander et al., 2015; Costa et al., 2018; Romagnoli et al., 2018; Takagi et al., 2019) endogenous gene tagging (Lander et al., 2016b, 2017; Soares Medeiros et al., 2017; Costa et al., 2018), gene complementation (Chiurillo et al., 2017), and gene knock-in (Chiurillo et al., 2019). These studies have been recently reviewed in Lander and Chiurillo (2019).

Control of gene expression can be achieved at the transcriptional, translational or posttranslational levels (Ganesan et al., 2016). In the case of the related trypanosomatid *Trypanosoma brucei*, the fastest method for the generation of conditional mutants is the use of RNA interference (Alibu et al., 2005). This pathway, however, is absent in *T. cruzi* (Darocha et al., 2004). Integration of a tetracycline-regulated extra copy of the gene of interest to allow the knockout of the endogenous alleles in a cell line stably expressing a *tet* repressor and the T7 RNA polymerase (inducible knockout) has also been successfully employed in *T. brucei* (Clayton, 1999). Efforts to develop a similar method for *T. cruzi* have mostly failed until now (Darocha et al., 2004; Burle-Caldas Gde et al., 2015). In contrast to the control of a reporter gene expression over a range of four orders of magnitude in response to tetracycline in *T. brucei*, relatively high expression levels of the gene was detected in *T. cruzi* in the absence of tetracycline and little increase was detected after tetracycline addition [reviewed by (Burle-Caldas Gde et al., 2015)]. Inducible systems using destabilization domains of dihydrofolate reductase (DDD), or the rapamycin binding protein (ddFKBP), were only used to either create suicidal *T. cruzi* strains (Ma et al., 2015), or did not mediate the efficient knockdown of the genes (Burle-Caldas Gde et al., 2015). Inducible expression of dimerizable CRE recombinase (DiCRE system) was also tried in *T. cruzi* but has been only used for removal of exogenous selectable markers from the parasite's genome with limited success (Kangussu-Marcolino et al., 2014).

We recently reported the use of an alternative method for downregulation of gene expression in *T. brucei*, mediated at the mRNA level, using riboswitches (Cruz-Bustos et al., 2018b), which are naturally occurring self-cleaving RNAs (ribozymes) that can be modified to respond to ligands (Winkler et al., 2004). We used the *glmS* gene from *Bacillus subtilis*, which can control reporter gene expression in response to exogenous glucosamine in other eukaryotes, such as *Saccharomyces cerevisiae* (Watson and Fedor, 2011) and *Plasmodium falciparum* (Prommana et al., 2013). The *glmS* gene encodes the enzyme glutamine-fructose 6-phosphate amidotransferase that uses fructose 6-phosphate and glutamine to generate glucosamine 6-phosphate (GlcN6P). A conserved element in the 5′-unstranslated region of this gene acts, when transcribed into RNA, as a self-cleaving riboswitch stimulated by glucosamine 6-phosphate (GlcN6P) (Winkler et al., 2004). When this conserved element is inserted in the 5′-UTR or the 3′-UTR of a gene of interest the self-cleaving RNA motif will silence it when in the presence of GlcN6P produced within the cells. Addition of glucosamine to the culture medium stimulates this activity through the endogenous generation of GlcN6P. A mutant *glmS* gene whose RNA has no self-cleaving activity (*M9*) can be used as negative control (Winkler et al., 2004). Tagging only one allele of the gene of interest (GOI) in *T. brucei* with *glmS* was sufficient to down-regulate gene expression at the mRNA level, and in some cases, produce phenotypic changes (Cruz-Bustos et al., 2018b). Since this technique requires the endogenous tagging of the genes that are targeted for down-regulation, our recent development of C-terminal endogenous tagging of genes in *T. cruzi* using CRISPR/Cas9 (Lander et al., 2016b, 2017) made this approach feasible.

In this work, we report the use of the *glmS* ribozyme for silencing the expression of endogenous genes without the need to add glucosamine to the medium, suggesting that *T. cruzi* produces enough levels of endogenous GlcN6P to stimulate the *glmS* ribozyme activity under normal growth conditions. This method could be useful to validate potential drug targets in this parasite.

## Results

### Effect of Glucosamine on Growth of Epimastigotes

We first tested whether glucosamine has any effect on parasite growth. Figure S1 shows the effects of 0–30 mM glucosamine added to a modified SDM-79 medium. This medium, which has been used to grow *T. cruzi* (Hasne et al., 2010), has a known concentration of glucosamine (1 mM) that could be varied to stimulate the riboswitch. We found that 10 mM was the maximum concentration that did not affect growth during the evaluated period and used that concentration in subsequent experiments.

### Downregulation of the Expression of *T. cruzi* Glycoprotein 72 (TcGP72)

**Figure 1:**
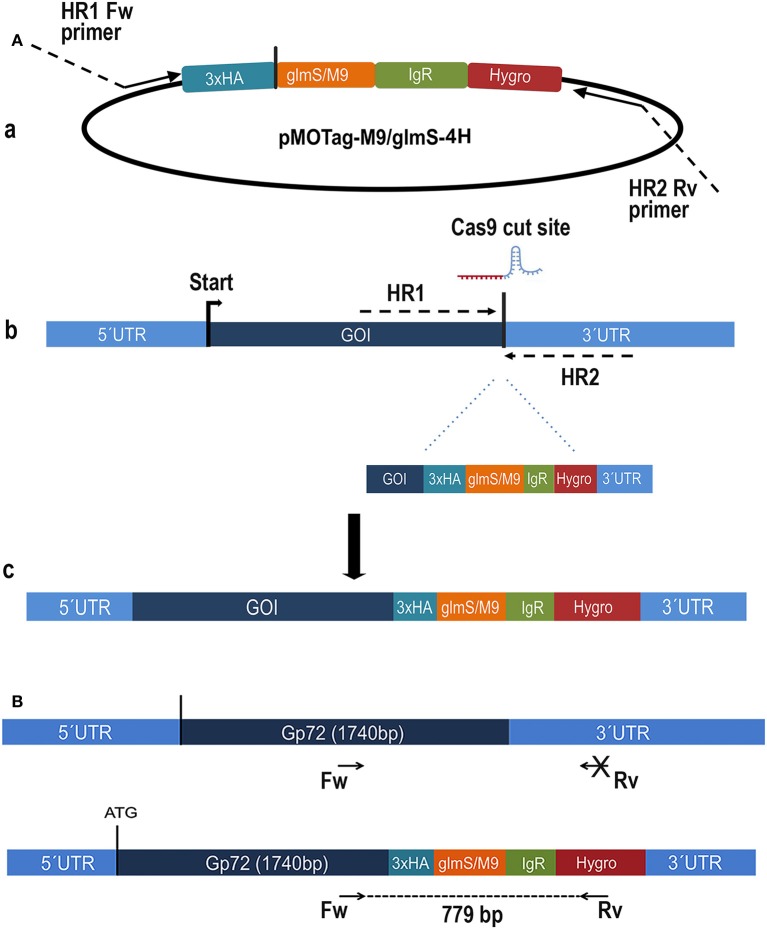
Schematic representation of the strategy used in *Trypanosoma cruzi*. **(A)** (a) pMOTag-4H-M9/glmS vector map. HR1 Fw, and HR2 Rv, ultramers indicate oligonucleotides used to amplify the DNA donor. (b) A doubled strand break was produced in the gDNA by Cas9 targeted by the sgRNA downstream of the STOP codon of the gene of interest (GOI), both expressed from 3′end-sgRNA/Cas9/pTREX-n plasmid. Homologous directed repair was induced co-transfecting epimastigotes with the DNA donor cassette, containing homologous regions to the GOI 3′ end (dark blue) and to the GOI 3′UTR (light blue). (c) Integration of 3xHA, *M9/glmS* and antibiotic resistance genes at 3′end of GOI by homologous recombination. **(B)** Diagram representing the positions of the primers (arrows) used to verify the integration of the donor DNA at the 3′ end of *TcGP72* ORF.

**Figure 2:**
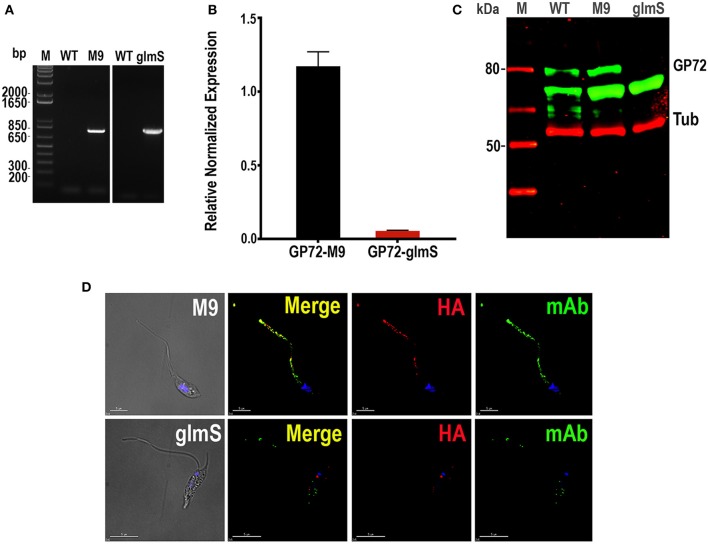
Integration of *glmS/M9* ribozyme sequences into the *TcGP72* gene. **(A)** PCR analysis using gDNA isolated from WT and *TcGP72-M9/glmS* (M9, glmS) cell lines. A DNA fragment of 779 bp was amplified in *3xHA-glmS/M9*-tagged epimastigotes, whereas the band is absent in WT cells. **(B)** qRT-PCR shows down-regulation of *TcGP72*. **(C)** Western-blot analysis of TcGP72-*M9/glmS* cell lines. Antibody WIC 29.26 labels a band of 72 kDa in *TcGP72-3xHA*-*M9*- but not in *TcGP72-3xHA*-*glmS*-tranfected cells. Anti-tubulin antibody (Tub) was used as loading control. Antibodies are indicated on the right side of the blots, and molecular weights (kDa) are on the left side. **(D)** Immunofluorescence microscopy of *TcGP72-3xHA-M9- and TcGP72-3xHA-glmS*-transfected epimastigotes. There is co-localization in the flagellum of anti-HA and WIC 29.26 antibodies in *TcGP72-3xHA-M9*-transfected epimastigotes (*merge*) while the flagellum is detached and has little labeling with anti-HA and reduced labeling with WIC 29.26 in *TcGP72-3xHA-glmS*-expressing cells. DIC, differential interference contrast microscopy. Scale bars = 5 μm.

We first used the CRISPR/Cas9 system (Lander et al., 2016b, 2017) for endogenously tagging *T. cruzi* glycoprotein 72 (*TcGP72*) with the active (*glmS*) or inactive (*M9*) ribozyme following the strategy shown in Figure 1. TcGP72 is a glycoprotein responsible for adhesion of the flagellum to the cell body and is not essential for the parasite survival (Cooper et al., 1993). We co-transfected a specific GP72-3'end-sgRNA/Cas9/pTREX-n construct with a specific DNA donor molecule amplified from the pMOTag-*M9/glmS*-4H vectors (Cruz-Bustos et al., 2018b), as described under Experimental Procedures and grew the cells initially in a modified SDM-79 medium. However, when glucosamine addition was shown to be unnecessary to downregulate *TcGP72* expression further experiments were done in regular LIT medium. We obtained G418/hygromycin resistant cells after 3 weeks under selective pressure. Transfectants were analyzed by PCR, using gDNA isolated from them, and a specific primer set to distinguish between the wild type and the tagged cell lines (Figure 1). After 21 days in culture it was possible to confirm the presence of the tagged gene (band of 779 bp) in *TcGP72-3xHA*-*glmS* (glmS) and *TcGP72-3xHA*-*M9* (M9) transfectants, but not in wild type (WT) parasites (Figure 2A). Insertion of *glmS* and *M9* constructs at the 3′UTR of *GP72* gene was confirmed by sequencing (Sequences S1, S2, respectively). qRT-PCR showed complete downregulation of *TcGP72* expression (Figure 2B). Western blot analysis using monoclonal antibody WIC 29.26 showed disappearance of the 72-KDa band after 45 days in culture compared to WT and *TcGP72-3xHA-M9*-transfectants (Figure 2C). As reported before (Cooper et al., 1993), this antibody recognizes the glycan epitope in additional proteins. Figure 2D shows the presence of parasites with flagellar detachment and greatly reduced labeling with monoclonal antibody WIC 29.26. More than 90% of the cells showed this phenotype.

**Figure 3:**
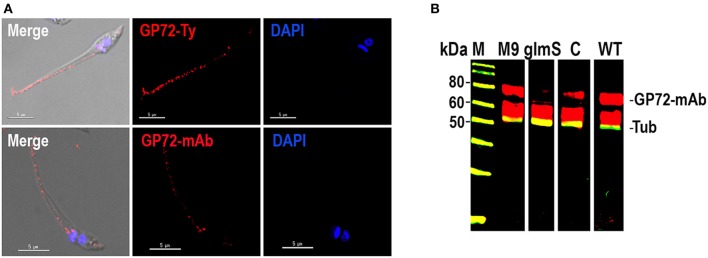
Complementation of *TcGP72-3xHA-glmS*. **(A)** Immunofluorescence microscopy of *TcGP72-3xHA-glmS* epimastigotes complemented with an exogenous copy of *TcGP72* gene. Labeling of the flagellum was detected with anti-Ty and WIC 29.26 antibodies, while no flagellar detachment was observed. Bars = 5 μm. **(B)** Western-blot analysis of total protein extracts of *TcGP72-3xHA-M9* (M9)*, TcGP72-3xHA-glmS* (glmS), *TcGP72-3xHA-glmS* complemented with *TcGP72-Ty* (lane C) and wild type (WT) epimastigotes, using WIC 29.26 antibodies. Anti-α-tubulin antibodies (Tub) were used as a loading control.

To exclude the possibility of off target effects, we investigated whether an exogenous *TcGP72* gene could complement the *TcGP72* knock down epimastigotes. We found that the exogenous gene (with changes in the PAM sequence to prevent disruption by CRISPR/Cas9) bearing a Ty epitope was targeted to the epimastigote flagellum, as shown in Figure 3A. Western blot analysis showed recovery of the labeling with antibody WIC 29.96 in complemented epimastigotes as compared to *TcGP72-3xHA*-*glmS* transfectants (Figure 3B). In conclusion, CRISPR/Cas9-mediated endogenous C-terminal tagging of *T. cruzi GP72* with *glmS* was successful in silencing the gene without the need to add glucosamine to the medium. These results suggest that *T. cru*zi produces high levels of endogenous GlcN6P and that these levels are sufficient to stimulate the *glmS* ribozyme activity under normal growth conditions.

### Downregulation of the Expression of *T. cruzi* Vacuolar Proton Pyrophosphatase (TcVP1)

**Figure 4:**
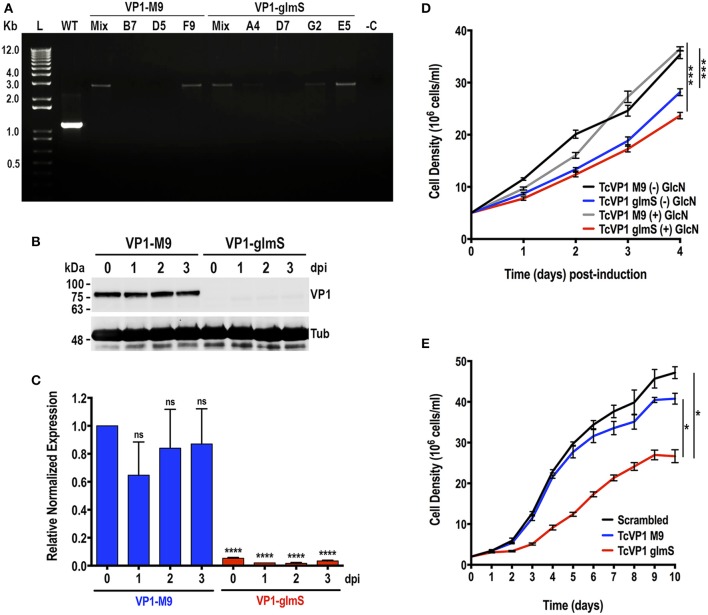
Integration of *glmS/M9* ribozyme sequences into the *TcVP1* gene. **(A)** PCR analysis using gDNA isolated from WT and *TcVP1-M9/glmS* mixed (Mix) and clonal populations (*TcVP1-M9*, clones B7, D5, and F9; *TcVP1-glmS*, clones D7, G2, and E5). The primer set used (Table S1, primers 11 and 12) generates a band of 1,144 bp in wild type parasites (WT) and a band of 2,758 bp in *TcVP1*-tagged (*M9* or *glmS*) epimastigotes. Transfectant mixed and clonal populations were analyzed from both tagged versions of *TcVP1*, and in both cases the band of 2,758 bp was detected in the originally transfected cell lines (Mix) and in some of the clones. Clones F9 (*TcVP1-M9*) and E5 (*TcVP1-glmS*) were chosen for further phenotypic analysis. **(B)** Western-blot analysis of *TcVP1-M9* and *TcVP1-glmS* cell lines. Anti TcVP1 monoclonal antibody labels a band of 85 kDa in *TcVP1-3xHA*-*M9*- but not in *TcVP1-3xHA*-*glmS*- epimastigotes. Expression of TcVP1 was monitored during 3 days after addition of 10 mM glucosamine to the medium (days post induction, dpi). Anti-tubulin antibody (Tub) was used as loading control. Antibodies are indicated on the right side of the blots, and molecular weights (kDa) are on the left side. **(C)** qRT-PCR analysis of *TcVP1-M9* and *TcVP1-glmS* cell lines from 0 to 3 days post induction (dpi). **(D)** Growth *in vitro* of *TcVP1-M9* and *TcVP1-glmS* epimastigotes cultured with [(+) GlcN] or without [(–) GlcN] 10 mM glucosamine. **(E)** Growth *in vitro* of scrambled, *TcVP1-M9* and *TcVP1-glmS* epimastigotes cultured in LIT medium until reaching the stationary phase, without addition of glucosamine. In **(C)**, qRT-PCR data analysis was performed using one-way ANOVA with multiple comparisons (*n* = 3; ****p < 0.0001; ns, no significant). In **(D,E)**, one-way ANOVA with multiple comparisons was applied to growth rates calculated from each growth curve (*n* = 3; *p <0.05; ***p <0.001).

The *T. cruzi* vacuolar proton pyrophosphatase (TcVP1) is an electrogenic proton pump mainly localized to acidocalcisomes (Scott et al., 1998; Lander et al., 2016b), where it maintains their acidity. In *T. brucei*, RNAi experiments have shown that the enzyme is essential for normal growth of procyclic and bloodstream forms *in vitro* (Lemercier et al., 2002). We followed the same procedure used to downregulate the expression of *TcGP72*. Chimeric sgRNA was cloned into *Cas9/pTREX-n* vector and co-transfected with DNA donor containing *glmS* ribozyme sequence or its *M9* inactive version into *T. cruzi* epimastigotes. Donor DNAs were amplified from *pMOTag-glmS-4H* and *pMOTag-M9-4H* vectors, respectively (Cruz-Bustos et al., 2018b). Transfectant parasites were obtained after 3 weeks of selection with G418 and hygromycin. We then obtained clonal populations from both *TcVP1-3xHA-M9* and *TcVP1-3xHA-glmS* tagged cell lines by serial dilutions. We confirmed *TcVP1* tagging in mixed and clonal populations by PCR (Figure 4A). We chose *TcVP1-3xHA-M9*, clone F9, and *TcVP1-3xHA-glmS*, clone E5 for further experiments. TcVP1 downregulation in *glmS*-tagged but not in *M9*-tagged epimastigotes was confirmed by western blot analysis using monoclonal antibodies anti-TcVP1 in the absence of 10 mM glucosamine (day 0) or at days 1, 2, and 3 after addition of glucosamine to the medium (Figure 4B). *TcVP1* expression was also evaluated at the RNA level by quantitative RT-PCR under the same conditions (Figure 4C). Downregulation of the gene in TcVP1-*glmS* epimastigotes in the absence of glucosamine (day 0) or at days 1, 2, and 3 post-induction (dpi) was confirmed, although basal levels of *TcVP1* expression were detected at all time points in TcVP1-*glmS* cells, relative to TcVP1-*M9* uninduced epimastigotes (day 0). No significant differences were observed in TcVP1-*M9* parasites at days 1, 2, and 3 post induction relative to day 0 (uninduced) (Figure 4C). We also evaluated the growth *in vitro* of *TcVP1-M9-* and *TcVP1-glmS*-tagged epimastigotes (Figures 4D,E). Growth of *TcVP1-glmS*-expressing epimastigotes in LIT medium was significantly affected as compared with the growth of *TcVP1-M9*-tagged cells but the presence of 10 mM glucosamine did not change the growth rate of these mutants (Figure 4D). The growth of these cell lines was then monitored for a longer period (10 days) in LIT medium, including a cell line expressing Cas9 and a scrambled sgRNA as control (*Scrambled*) (Figure 4E). Again, the results show a significant lower growth of *TcVP1-glmS*-expressing epimastigotes as compared with those expressing *TcVP1-M9* or a scrambled sgRNA, thus confirming the results observed with downregulation of *TcGP72*.

**Figure 5:**
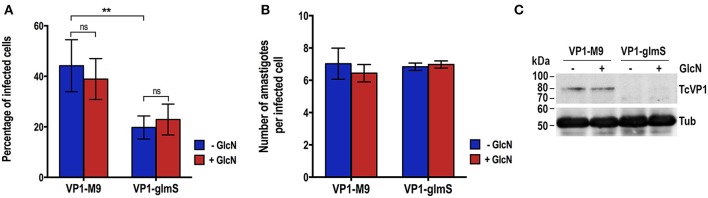
Invasion and intracellular replication of *TcVP1-M9* and *TcVP1-glmS* cell lines. **(A)** *TcVP1-M9* and *TcVP1-glmS* trypomastigote infection of Vero cells. There was a significant difference in the percentage of infected Vero cells but not in the number of intracellular amastigotes per infected host cell observed 48 h post infection **(B)**. Values are mean ± s.d.; *n* = 3; **p < 0.01; *ns*, not significant (Two-way ANOVA with multiple comparisons test). **(C)** Western-blot analysis of *TcVP1-M9* and *TcVP1-glmS* trypomastigotes. Anti TcVP1 antibody labels an expected band of 85 kDa in *TcVP1-3xHA*-*M9*- but not in *TcVP1-3xHA*-*glmS*- epimastigotes. Tissue culture-derived trypomastigotes collected at day 6 post-infection of Vero cells were induced with 10 mM glucosamine (GlcN) overnight at 4°C. After induction, trypomastigotes were used for infection of Vero cells and 1 × 10^7^ trypomastigotes were reserved for protein extraction. Proteins were analyzed by western blot using TcVP1 mAb (1:2,000). Anti-tubulin antibody (Tub) was used as loading control. Antibodies are indicated on the right side of the blots, and molecular weights (kDa) are on the left side.

We induced the differentiation of *TcVP1*-*glmS* and *TcVP1-M9* epimastigotes to metacyclic forms and infected Vero cells as described in Experimental procedures. Once enough culture-derived trypomastigotes were obtained we used them to test their ability to infect Vero cells and replicate intracellularly as amastigotes. Figure 5 shows that the ability of *TcVP1-glmS* trypomastigotes to infect tissue-culture cells, but not the amastigote replication, was significantly impaired, as compared with that of *TcVP1-M9* parasites. The presence of added glucosamine had no effect in either invasion or replication, thus confirming the results with epimastigotes in both *TcGP72*-*glmS* and *TcVP1*-*glmS* cells. Downregulation of TcVP1 in the *TcVP1-glmS* but not in the *TcVP1-M9* trypomastigotes used for the invasion assays was confirmed by western blot analysis (Figure 5C).

## Discussion

CRISPR/Cas9-mediated endogenous C-terminal tagging of *TcGP72* and *TcVP1* with *glmS*, but not with *M9*, was successful in knocking down the gene expression without the addition of glucosamine to the medium. These results suggest that *T. cru*zi produces high levels of endogenous glucosamine 6-phosphate and that these levels are sufficient to stimulate the *glmS* ribozyme activity under normal growth conditions. In this regard, it has been found that *T. cruzi* (TcCLB.506507.10), as well as *Leishmania major* (LmjF32.3260), possesses an N-acetyl glucosamine 6-phosphate deacetylase (NAGD) that generates glucosamine 6-phosphate while *T. brucei* lacks this enzyme (Naderer et al., 2010). It is tempting to speculate that this enzyme could be involved in the production of high endogenous levels of glucosamine 6-phoshate in *T. cruzi*. However, it is important to indicate that the method, as it has been developed until now, could be useful to obtain knockdowns of essential genes in *T. cruzi*. If the gene is essential, and depending on the ribozyme used, the cells will die (*glmS* transfectants) or survive (*M9* transfectants). This method could then be used to validate the essentiality of potential targets. In addition, as the *glmS* ribozyme acts at the mRNA level, it is possible to detect basal levels of expression in the transfectants, thus allowing the selection of parasites expressing *glmS*-tagged essential genes, as shown in Figure 4C for *TcVP1* gene. C-terminal endogenous tagging also facilitates localization studies of the genes of interest.

One advantage of using our previously reported CRISPR/Cas9 methodology (Lander et al., 2016b, 2017) for tagging genes with *glmS* ribozyme, is that the constitutive expression of Cas9 and the sgRNA in the presence of a DNA donor conferring antibiotic resistance, allows tagging both alleles of the gene using a single resistance marker, as previously shown (Lander et al., 2015, 2018; Chiurillo et al., 2017, 2019; Cruz-Bustos et al., 2018a; Bertolini et al., 2019). In this way, the downregulation efficiency is significantly improved because only one transfection is necessary to tag and inactivate both alleles of the gene.

It has been reported that in *S. cerevisiae* (Meaux and Van Hoof, 2006) and in *P. falciparum* (Prommana et al., 2013) the ribozyme-cleaved mRNA 5′ fragment separated from its polyA tail could be degraded by the 3′exosome. This pathway has also been reported to be active in trypanosomatids (Estevez et al., 2001). When the *glmS* ribozyme is in the 5′UTR, mRNA cleavage would separate the mRNA from its 5′cap structure and the de-capped mRNA would be degraded by the 5′-3′-exonucleases, which are also present in trypanosomatids (Li et al., 2006).

Interestingly, our studies revealed that, in contrast to what occurs with TbVP1 in *T. brucei* (Lemercier et al., 2002), TcVP1 is not essential for the viability of epimastigotes or the infective stages of *T. cruzi*, although is important for normal growth of epimastigotes in rich medium, and for trypomastigote invasion of host cells. TcVP1 is mostly localized to acidocalcisomes where it contributes to the acidification of the organelle together with a vacuolar proton ATPase (Docampo et al., 1995; Scott et al., 1998).

In conclusion, the CRISPR/Cas9/riboswitch method developed in this work will enable the downregulation of gene expression in *T. cruzi*, and potentially in *Leishmania* spp. in which endogenous gene tagging using CRISPR/Cas9 has been achieved (Lander et al., 2016a,b).

## Experimental Procedures

### Culture Methods

*Trypanosoma cruzi* Y strain epimastigotes were cultured in liver infusion tryptose (LIT) medium (Bone and Steinert, 1956) containing 10% heat-inactivated fetal bovine serum at 28°C. The endogenously tagged cell line was maintained in medium containing 250 μg/ml G418 and 150 μg/ml of hygromycin. For initial experiments to test the effect of glucosamine, epimastigotes were also cultured in medium SDM-79, without the addition of D-glucosamine and supplemented with hemin (7.5 μg/mL), 200 μM putrescine, and 10% heat-inactivated fetal bovine serum (Hasne et al., 2010; Jimenez and Docampo, 2015). Cell growth was determined by counting cells in a Neubauer chamber.

### Chemicals and Reagents

The pMOTag4H vector was a gift from Dr. Thomas Seebeck (University of Bern, Bern, Switzerland). Monoclonal antibody WIC 29.26 was a gift from Dr. George A.M. Cross (Rockefeller University). Wild type and *M9* mutated *Bacillus subtilis glmS* ribozymes were gifts from Dr. Vasant Muralidharan (University of Georgia). Monoclonal antibody BB2 against *the S. cerevisiae* Ty1 virus-like particle was a gift from Dr. R. Drew Etheridge (University of Georgia). Monoclonal antibody against *T. cruzi* vacuolar H^+^-pyrophosphatase (TcVP1) was described before (Seufferheld et al., 2004). GoTaq DNA polymerase and T4 DNA ligase were from Promega. Antarctic phosphatase, restriction enzymes, and Q5 high fidelity DNA polymerase were from New England Biolabs (Ipswich, MA). Fluoromount-G was from SouthernBiotech (Birmingham, AL). Pierce BCA protein assay, Hygromycin B, BenchMark prestained protein ladder, BenchMark protein ladder, MagicMark™ XP Western Protein Standard, Alexa-conjugated secondary antibodies, and HRP-conjugated secondary antibodies were from Thermo Fisher Scientific. Anti-HA high affinity rat monoclonal antibody (clone 3F10) was purchased from Roche Applied Science. IRDye-conjugated secondary antibodies were from LI-COR Biosciences (Lincoln, NE). Benzonase nuclease was from Novagen (EMD Milli- pore, Billerica, MA). Nitrocellulose membranes were from Bio-Rad. The primers were purchased from Integrated DNA Technologies. TRI® reagent, anti-tubulin monoclonal antibody, G418, mammalian cell protease inhibitor mixture (P8340), other protease inhibitors, and all other reagents of analytical grade were from Sigma-Aldrich.

### Molecular Constructs

The pMOTag4H vector designed for endogenous C-terminal tagging of *T. brucei* (Oberholzer et al., 2006) was used to construct the template plasmids for DNA donor amplification. This vector contains a 3xHA tag and the hygromycin resistance marker. Wild-type and *M9* mutated *Bacillus subtilis glmS* ribozyme sequences (Winkler et al., 2004) were used to generate the pMOTag-*glmS*-4H and pMOTag-*M9*-4H vectors (Addgene plasmids #106378 and #106379) (Cruz-Bustos et al., 2018b). For CRISPR/Cas9-mediated endogenous C-terminal tagging in *T. cruzi* we used the Cas9/pTREX-n vector (Addgene plasmid #68708) (Lander et al., 2015) to clone a specific single guide RNA (sgRNA) sequence targeting the 3′end of *TcGP72* (TcCLB.50956120) and *TcVP1* (TcCLB. 510773.20) genes. The sgRNAs were amplified by PCR (Table S1, primers 1–3), using pUC_sgRNA plasmid as template (Addgene plasmid #68710**)** (Lander et al., 2015). We co-transfected the GP72-3′end-sgRNA/Cas9/pTREX-n or VP1-3′end-sgRNA/Cas9/pTREX-n construct with the specific DNA donor template for tagging the 3′ end of each gene, right upstream the stop codon, with a 3xHA tag sequence and the *glmS/M9* ribozyme amplified from pMOTag-glmS-4H and pMOTag-M9-4H vectors, respectively, as previously described (Lander et al., 2016b; Cruz-Bustos et al., 2018b) (Table S1, primers 4–7). sgRNA correct orientation in Cas9/pTREX-n vector was determined by PCR (Table S1, primers 1, 2, and 8) and sequencing using primer 8. Endogenous tagging of *TcVP1* and *TcGP72* was confirmed by PCR (Table S1, primers 9–12). A cell line expressing Cas9 and a scrambled sgRNA (Lander et al., 2015) was used a control for growth experiments *in vitro*.

### Complementation of TcGP72-KO Cells

To revert the phenotype exhibited by *TcGP72-g3xHA-glmS* epimastigotes we used an exogenous *TcGP72* gene to complement the mutants. Following a PCR strategy, we eliminated the PAM sequence (TGG–TGT) specific for the *TcGP72*-sgRNA used to obtain the *TcGP72-g3xHA-glmS* cells, therefore avoiding constitutively expressed Cas9 to target the inserted sequence (Table S1, primers 13 and 14). The PCR product was cloned into pTREX-p vector (Chiurillo et al., 2017), which confers resistance to puromycin, by XbaI and XhoI restriction sites. We also included a C-terminal Ty1 tag in the reverse primer 14 (Table S1) in order to detect the overexpressed protein using anti-Ty1 antibody.

### Cell Transfection

*Trypanosoma cruzi* epimastigotes were grown to a density of 1–2 × 10^7^ cells/ml, washed once with cold PBS, pH 7.4, and resuspended in ice-cold Cytomix (120 mM KCl, 0.15 mM CaCl_2_, 10 mM K_2_HPO_4_, 2 mM EDTA, 5 mM MgCl_2_, pH 7.6) at a density of 10^8^ cells/ml in electroporation buffer. Transfections were carried out in a 4-mm cuvette with 25 μg of plasmid DNA and 25 μg of DNA donor, using the Bio-Rad Gene Pulser Xcell electroporator set at 1.5 kV and 25 μF with three pulses, allowing at least 1 min for cells to recover in ice between pulses, and then incubated at room temperature for 15 min. Parasites were recovered in 5 ml of LIT supplemented with 20% fetal bovine serum at 28°C and after 24 h in culture, geneticin (G418), and hygromycin B were added to a final concentration of 250 and 150 μg/ml, respectively.

### Western Blot Analyses

Electrophoresed proteins were transferred to nitrocellulose membranes using a Bio-Rad transblot apparatus for 1 h at 100 V at 4°C. Following transfer, the membrane blots were blocked with 5% non-fat dry milk in PBS containing 0.1% Tween-20 (PBS-T) overnight at 4°C. Blots were probed with primary antibody (WIC 29.26 monoclonal antibody (1:1,000), anti-Ty1 monoclonal antibody (1:1,000), anti TcVP1 monoclonal antibody (1:2,000) or anti-tubulin monoclonal antibody [1:40,000)] for 1 h, at RT. After washing three times with PBS-T, the blots were incubated with goat anti-rabbit antibody (1:20,000) or goat anti-mouse antibody (1:20,000). The membranes were washed three times with PBS-T, and western blot images were processed and analyzed using the Odyssey infrared system software (LICOR Biosciences) or a ChemiDoc™ Imaging System (Bio-Rad).

### Immunofluorescence Analyses

Epimastigotes in log phase were washed once with PBS at room temperature and fixed with 4% paraformaldehyde in PBS for 30 min at room temperature. The cells were allowed to adhere to poly-L-lysine-coated coverslips and then permeabilized for 3 min with 0.3% Triton X-100. Permeabilized cells were blocked with PBS containing 3% BSA, 1% fish gelatin, 50 mM NH_4_Cl, and 5% goat serum 1 h at room temperature. Then cells were incubated with the primary antibody (1:100 rat anti-HA tag, 1:500 anti-Ty1 monoclonal antibody and 1:100 mouse WIC 29.26 diluted in PBS (pH 7.4) for 1 h at room temperature. The cells were washed three times with in PBS (pH 7.4) and then incubated for 1 h at room temperature in the dark with Alexa Fluor 488-conjugated goat anti-mouse and Alexa Fluor 546-conjugated goat anti-rat (1:1,000). Following incubation with the secondary antibody, the cells were washed five times in PBS and once in water and then mounted on slides. DAPI (5 μg/ml) was included in the Fluoromount-G mounting medium to stain DNA. Controls were performed as described above using *M9*-tagged epimastigotes. Specimens were imaged using the Delta Vision Elite deconvolution microscope (Applied Precision).

### Metacyclogenesis

*Trypanosoma cruzi* epimastigotes were *in vitro* differentiated into infective metacyclic trypomastigotes by aging in LIT medium for 10 days at 28°C. Cultures were started at 5 × 10^6^ cells/mL and after 10 days, 1.5 mL aged culture was centrifuged at 1,000 × g for 7 min and resuspended in 5 mL RPMI supplemented with 20% FBS fresh. The complement in fresh FBS kills epimastigotes, whereas metacyclic trypomastigotes survive. Cells were examined under the microscope to confirm the presence of about 10% metacyclic trypomastigotes, and used immediately to infect Vero cells as described below.

### Host Cell Invasion and Intracellular Replication Assays

Gamma-irradiated (2,000 radiation-absorbed doses) Vero cells (4.5 × 10^5^ cells) were plated onto sterile coverslips in a 12-well plate and incubated overnight at 35°C, 7% CO_2_, in RPMI medium plus 10% fresh FBS. Tissue culture-derived trypomastigotes were incubated at 4°C overnight to allow amastigotes to settle from swimming trypomastigotes. Trypomastigotes from the supernatants of these collections were counted and used to infect the coverslips at a 10:1 ratio of parasites to host cells. At 4 h post-infection, coverslips were washed extensively with Hank's balanced salt solution, followed by PBS, pH 7.4, to remove any extracellular parasites. Coverslips were fixed immediately in 4% paraformaldehyde in PBS, pH 7.4, at 4°C for 30 min. Coverslips were washed once with PBS and mounted onto glass slides in Fluoromount-G containing 15 μg/ml DAPI, which stains host and parasite DNA. Coverslips were viewed on an Olympus BX60 microscope to quantify the number of host cells that contained intracellular parasites and the number of intracellular parasites per cell in randomly selected fields. Three hundred host cells were counted per sample in three independent experiments. To quantify amastigote replication, the following modifications were used: host cells were infected at a ratio of 10 parasites to one host cell, and coverslips were allowed to incubate for 48 h post-infection at 35°C, 7% CO_2_, prior to fixation and DAPI staining. Coverslips were mounted onto glass slides and analyzed by fluorescence microscopy. Amastigotes in infected cells were counted using a 100 × objective.

### Quantitative Real-Time PCR

Total RNA was isolated from trypanosomes using the TRI® reagent (Sigma) by following the manufacturer's instructions. The total RNA was treated with DNase I to remove genomic DNA contamination. cDNA synthesis was accomplished using the iScript cDNA synthesis kit (Bio-Rad) with 100 ng of total RNA used per reaction. Real-time PCR was done using a CFX96 Touch™ Real-Time PCR Detection System (Bio-Rad) and set up in hard-shell/clear 96-well PCR plates, in a final volume of 10 μl per reaction. The primers for gene amplification are listed in Table S1 (primers 15–24). Reaction mixtures contained 2 μl of sample DNA (100 ng/μl), 5 μl of a master mix iQ™ SYBR® Green Supermix (Bio-Rad) and 4 μl of nuclease-free water with primers at a final concentration of 300 nM. Activation of polymerase was performed at 95°C for 2 min. PCR cycling conditions included 39 cycles of denaturation at 95°C for 10 s, and annealing and extension at 60°C for 30 s (*GP72* gene) or 55 °C for 45 s (*TcVP1* gene). SYBR Green fluorescent emission was measured at the end of the elongation step. Subsequently, a melting curve program was applied with a continuous fluorescent measurement starting at 65°C and ending at 95°C (ramping rate of 0.1°C/s). In order to normalize the expression of the genes, we used primers for *P0* and *L3* housekeeping genes (Table S1, primers 15–18 used for *GP72* normalization) and α*-Tubulin* (Table S1, primers 21 and 22 used for *TcVP1* normalization) from *T. cruzi*. Relative quantification normalized to reference genes was performed according to the ΔC_T_ method and all the assays were performed at least three times.

### Statistical Analysis

All values are expressed as means ± s.d. Significant differences between treatments were compared using the tests indicated in the figure legends. Differences were considered statistically significant at *P* < 0.05, and *n* refers to the number of independent biological experiments performed. All statistical analyses were conducted using GraphPad Prism 5 (GraphPad Software, San Diego, CA).

## Data Availability Statement

The raw data supporting the conclusions of this article will be made available by the authors, without undue reservation, to any qualified researcher.

## Author Contributions

NL, TC-B, and RD designed the experiments and analyzed the data. NL and TC-B conducted the experiments. RD wrote the majority of the manuscript with specific sections contributed by NL and TC-B. RD supervised the work and contributed to the analysis of the experiments.

### Conflict of Interest

The authors declare that the research was conducted in the absence of any commercial or financial relationships that could be construed as a potential conflict of interest.

## Supplementary Material

Figure S1 Effect of different glucosamine concentrations on growth of epimastigotes in SDM-79 medium. Values are means ± s.d. of *n* = 3.

Table S1 Primers used in this work.

Sequence S1 GP72-glmS locus sequenced with primer 9 (Table S1).

Sequence S2 GP72-M9 locus sequenced with primer 9 (Table S1).
