# Supplementary figures and images for: A CRISPR/Cas9-riboswitch-Based Method for Downregulation of Gene Expression in Trypanosoma cruzi
Source: Front Cell Infect Microbiol. 2020 Feb 27;10:68. doi: 10.3389/fcimb.2020.00068 (PMC7056841; doi:10.3389/fcimb.2020.00068)

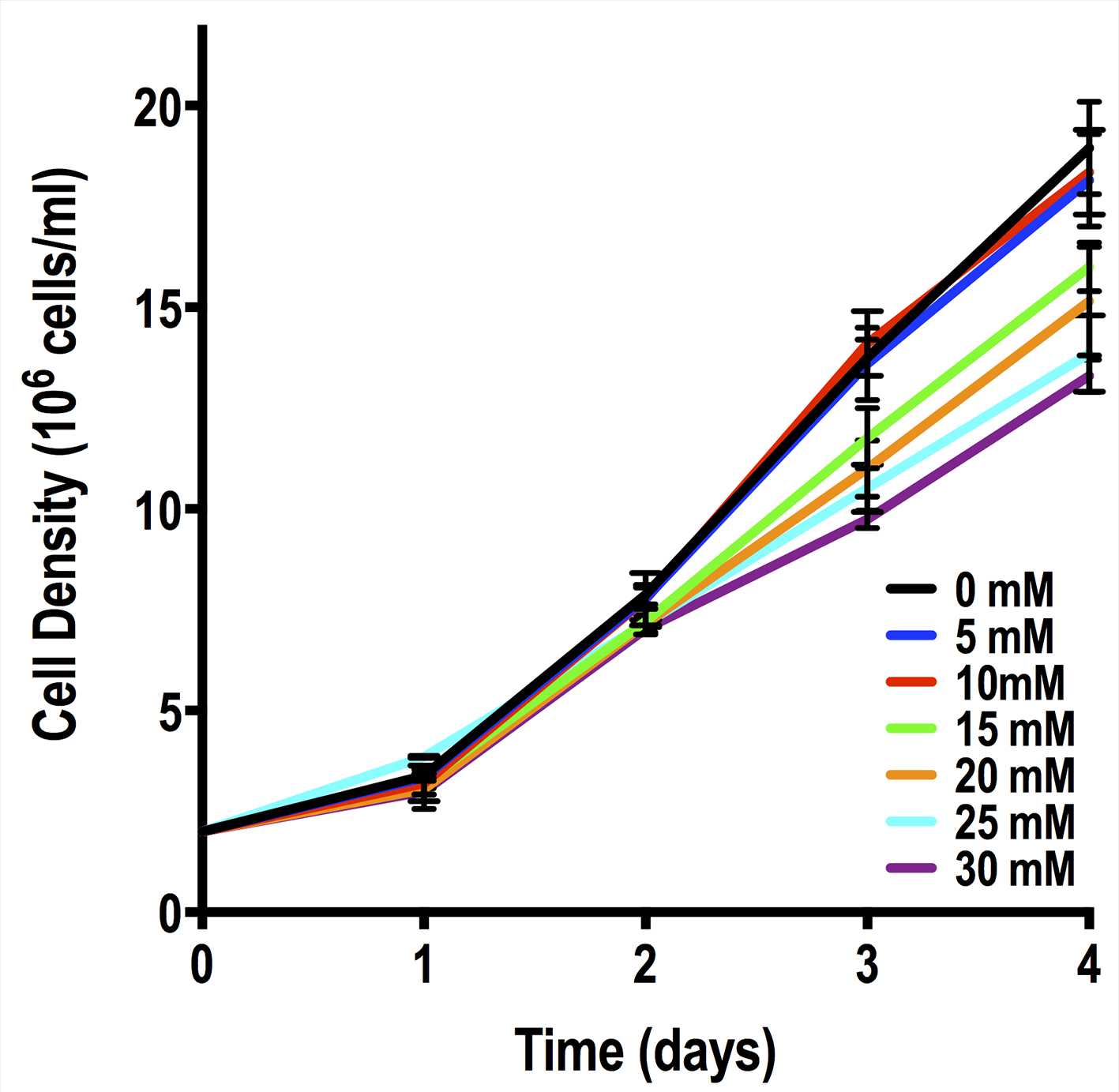

Supplement: Figure S1 — Effect of different glucosamine concentrations on growth of epimastigotes in SDM-79 medium. Values are means ± s.d. of n = 3. [file Image_1.TIF]
