# Supplementary material for: A CRISPR/Cas9-riboswitch-Based Method for Downregulation of Gene Expression in Trypanosoma cruzi
Source: Front Cell Infect Microbiol. 2020 Feb 27;10:68. doi: 10.3389/fcimb.2020.00068 (PMC7056841; doi:10.3389/fcimb.2020.00068)
Supplement: Table S1 — Primers used in this work. [file Table_1.pdf]

Table S1. Primers used in this study

Number	*Primer*	*Sequence (5'→ 3')*
1	**Fw-sgRNA-GP72**	GATCGGATCCGGGGACAAAATCTACATGGGGTTTTAGAGCTAGAAATAGC
2	**Fw-sgRNA-VP1**	GATCGGATCCAGTGTAACGCCGTGAACGCGGTTTTAGAGCTAGAAATAGC
3	**Rv-sgRNA**	CAGT*GGATCC*AAAAAAGCACCGACTCGGTG
4	**Fw-GP72-Ctag-ultramer**	GGCGGTAGTGTCCTCCGCGGTGCTTGGCGCCACAGGTATCGCACTCATTGCACTGATGGTTGG AAGTTCGGCGAACGTACGGAGCGCTGTGATTCTTGTTGGTACCGGGCCCCCCCTCGAG
5	**Rv-GP72-Ctag-ultramer**	CCCGTTGTTTTCACCTTCGCTTGCTTCTTGTATTGTTGGTTCCTTCTAATTTCAATTTCTGAAGTCA TTTCCCACCTTTCTCCGGGGCGGGGACAAAATTGGCGGCCGCTCTAGAACTAGTGGAT
6	**Fw-VP1-Ctag-ultramer**	CGCGCTGAACATTCTGATCAAACTGATGGCCATCATTTCGGTTGTCTTTGCGCCTGTCTTTGAGTC GCAGCTTGGCGGTATTATCATGCGGTACATTGAGGGTACCGGGCCCCCCCTCGAG
7	**Rv-VP1-Ctag-ultramer**	AGGCACAACCAGCGAGGAAAAAACAAGCGGGGGAGAAAATATGGCGACAGGCATAAATAAAAACATA AAAATAAAATAAAAAAAAACAAAAAACGTTCCTTGGCGGCCGCTCTAGAACTAGTGGAT
8	**Rv-HX1**	TAATTTCGCTTTCGTGCGTG
9	**Fw-GP72-Ctag-check**	CCCCCAGTATGCAACGACGTC
10	**Rv-pMOTag-Ctag-check**	CAGACGTCGCGGTGAGTTCAG
11	**Fw-VP1-Ctag-check**	CCACGAACATCATCTACGGC
12	**Rv-TcVP1-Ctag-check**	GTCGTTTTGTCCTCGTCGC
13	**Fw-GP72-Compl**	TTGTCTAGAATGTTTTCAAAAAGGACGTCGCCA
14	**Rv-GP72-Ty-Compl**	GATAAGCTTTCAATCGAGCGGGTCCTGGTTCGTGTGGACCTCCATGGGACAAACAAGAATCACA
15	**Fw-P0**	CCTTCTTCCAGGCACTGAAC
16	**Rv-P0**	AGTTGTCCACACGATCACCA
17	**Fw-L3**	TGGCTTTGTTGGCTACGGTAC
18	**Rv-L3**	CCACTGGCTCTTCTCCTTCTTTG
19	**Fw-GP72-RT**	GTACGGAGCGCTGTGATTCTTG
20	**Rv-GP72-pmotag-RT**	GTCGACTATGCGTAATCGGGCA
21	**Fw-Tubulin-RT**	GAGGGCATGGACGAGATG
22	**Rv-Tubulin-RT**	CTCCTCCTCGTCGAACTCA
23	**Fw-TcVP1-RT**	GATGATGCTCCGGAGAATGT
24	**Rv-TcVP1-RT**	GCTTTGGTGTAAATCCCACC
