# Supplementary material for: A CRISPR/Cas9-riboswitch-Based Method for Downregulation of Gene Expression in Trypanosoma cruzi
Source: Front Cell Infect Microbiol. 2020 Feb 27;10:68. doi: 10.3389/fcimb.2020.00068 (PMC7056841; doi:10.3389/fcimb.2020.00068)
Supplement: Sequence S1 — GP72-glmS locus sequenced with primer 9 (Table S1). [file Data_Sheet_1.pdf]

>GP72-glmS (766 bp)
NNNNNNNNNNNNNNNNNNNNNNCTGATGGTTGGAAGTTCGGCGAACGTACGGAGCGCTGTGATTCTTGTTGGT
ACCGGGCCCCCCCTCGAGTACCCTTACGATGTGCCTGATTACGCGTACCCATACGACGTGCCAGACTACGCAT
ACCCGTACGATGTGCCCGATTACGCATAGTCGACTGAGTAATTATAGCGCCCGAACTAAGCGCCCGGAAAAAG
GCTTAGTTGACGAGGATGGAGGTTATCGAATTTTCGGCGGATGCCTCCCGGCTGAGTGTGCAGATCACAGCCG
TAAGGATTTCTTCAAACCAAGGGGGTGACTCCTTGAACAAAGAGAAATCACATGATCTGTCGACAAAGTGTGA
CAACGTCGCACCATGTGTAGGTTTTCATTTATGTTCTTTCTTTCTTTCTTTTGTGAATTTGTTTTCTGTCTCA
AATGTTTTTAATTCGCTTGGGACCTATGTTTTTCTTGTTTTTTTGCTCACCCTTTGTGTAGGAGGCACCCTGT
CACGTCTGTGGTTGCGTGTATGCCTTCCTTCCCCTTATTCGCTTCTTCCTGTCGTGTCACACCTCTTTCTCCC
TCTCCCTTTCCGCCTTTTCTTTCAATCTTGTTTTCTCGACCAGCCCTACTAGAGGAGAAAGAATAGTAACCCT
TTCATCAAAGAAAATAGTTCAAACGAATTGCTAGCTTAAGCTTGATCTAGAACTAGTGATGAAAAAGCCTGAA
CTCACCGCGACGTCTGTCGAGAAGTTTCTGATCGAA
