# Supplementary material for: A CRISPR/Cas9-riboswitch-Based Method for Downregulation of Gene Expression in Trypanosoma cruzi
Source: Front Cell Infect Microbiol. 2020 Feb 27;10:68. doi: 10.3389/fcimb.2020.00068 (PMC7056841; doi:10.3389/fcimb.2020.00068)
Supplement: Sequence S2 — GP72-M9 locus sequenced with primer 9 (Table S1). [file Data_Sheet_2.pdf]

>GP72-M9 (872 bp)

NNNNNNNNNNNNNNNNNNNNNNNNNNNNNAAGTTCGGCGAACGTACGGAGCGCTGTGATTCTTGTTGGTACCG
GGCCCCCCCTCGAGTACCCTTACGATGTGCCTGATTACGCGTACCCATACGACGTGCCAGACTACGCATACCC
GTACGATGTGCCCGATTACGCATAGTCGACTGAGTAATTATCCCGCCCGAACTAAGCGCCCGGAAAAAGGCTT
AGTTGACGAGGATGGAGGTTATCGAATTTTCGGCGGATGCCTCCCGGCTGAGTGTGCAGATCACAGCCGTAAG
GATTTCTTCAAACCAAGGGGGTGACTCCTTGAACAAAGAGAAATCACATGATCTGTCGACAAAGTGTGACAAC
GTCGCACCATGTGTAGGTTTTCATTTATGTTCTTTCTTTCTTTCTTTTGTGAATTTGTTTTCTGTCTCAAATG
TTTTTAATTCGCTTGGGACCTATGTTTTTCTTGTTTTTTTGCTCACCCTTTGTGTAGGAGGCACCCTGTCACG
TCTGTGGTTGCGTGTATGCCTTCCTTCCCCTTATTCGCTTCTTCCTGTCGTGTCACACCTCTTTCTCCCTCTC
CCTTTCCGCCTTTTCTTTCAATCTTGTTTTCTCGACCAGCCCTACTAGAGGAGAAAGAATAGTAACCCTTTCA
TCAAAGAAAATAGTTCAAACGAATTGCTAGCTTAAGCTTGATCTAGAACTAGTGATGAAAAAGCCTGAACTCA
CCGCGACGTCTGTCGAGAAGTTTCTGATCGAAAAGTTCGACAGCGTCTCCGACCTGATGCAGCTCTCGGAGGG
CGAAGAATCTCGTGCTTTCAGCTTCGATGTAGGAGGGCGTGGATATGTCCTGCGGGTAAATAGCTGCGC
